# Global management of drug–drug interaction in patients with prostate cancer: international survey by Meet-URO group and ONCOassist

**DOI:** 10.1093/oncolo/oyag360

**Published:** 2026-09-16

**Authors:** Anna Amela Valsecchi, Michele Maffezzoli, Maria Concetta Cursano, Mattia Di Civita, Fiorenza Santamaria, Stefania Kingspergher, Elisa Zanardi, Giuseppe Procopio, Suleka Ashok, Lakshmi Ramachandran, Renita George, Kevin Bambury, Richard Bambury, Eoin O’Carroll, Giuseppe Luigi Banna, Massimo Di Maio, Daniele Santini

**Affiliations:** Department of Oncology, University of Turin, A.O.U. Città della Salute e della Scienza di Torino, Ospedale Molinette, 10126, Italy; Oncology Unit, University Hospital of Parma, Parma, 43126, Italy; Department of Oncology, Ospedale Ca’ Foncello, Treviso, 31100, Italy; Department of Experimental Medicine, Sapienza University of Rome, Rome, 00161, Italy; Department of Medical Oncology, Medical Oncology Unit, Sandro Pertini Hospital, Rome, 00157, Italy; Department of Experimental Medicine, Sapienza University of Rome, Rome, 00161, Italy; Ospedale San Maurizio, Bolzano, 39100, Italy; UO Clinica di Oncologia Medica, IRCCS Azienda Ospedaliera Metropolitana, Genoa, 16132, Italy; Department of Oncology, Genitourinary Medical Oncology, Fondazione IRCCS Istituto Nazionale Tumori Milano, Milan, 20133, Italy; ONCOassist, V93 T8K7, Ireland; ONCOassist, V93 T8K7, Ireland; ONCOassist, V93 T8K7, Ireland; ONCOassist, V93 T8K7, Ireland; ONCOassist, V93 T8K7, Ireland; ONCOassist, V93 T8K7, Ireland; Portsmouth Hospitals University NHS Trust, Portsmouth, PO6 3LY, United Kingdom; Faculty of Science and Health, School of Pharmacy and Biomedical Sciences, University of Portsmouth, Portsmouth, PO1 2UP, United Kingdom; Department of Oncology, University of Turin, A.O.U. Città della Salute e della Scienza di Torino, Ospedale Molinette, 10126, Italy; Department of Sciences and Medical-Surgery Biotechnologies, La Sapienza, Università di Roma, Rome, 04100, Italy

**Keywords:** survey, drug–drug interaction, Meet-URO, ONCOassist, prostate cancer, AIOM

## Abstract

**Background:**

Patients with prostate cancer (PC) frequently receive multiple medications for comorbidities, increasing the risk of drug–drug interactions (DDIs). Despite guidelines highlighting DDI relevance, data on real-world management are limited. This international survey aimed to characterize current practices, barriers, and needs in this setting.

**Materials and methods:**

A self-administered, multiple-choice online survey was sent to 3342 Italian oncologists (AIOM/Meet-URO members) and 19 139 global healthcare professionals via the ONCOassist app. A part of the survey included optional case studios completed only by Italian respondents.

**Results:**

Responses were received by 69 healthcare professionals in Italy and 1064 worldwide. Overall, DDIs were widely recognized as clinically relevant in PC care. Digital tools (online databases and smartphone applications) were the most frequently used resources for DDI assessment in both cohorts, whereas AI-based tools were limited. Italian oncologists reported limited on-site pharmacologist access (29%), while global respondents reported more frequent pharmacologist support (76%). Common barriers included time constraints, uncertain reliability of sources, and lack of training. When DDI checkers conflicted, Italian and global respondents favored pharmacologist consultation, although global users relied also on literature (31%) and clinical judgment (26%). Proposed solutions differed between cohorts: Italian clinicians emphasized the need for greater access to pharmacologists’ support, whereas the global cohort highlighted institutional training and educational initiatives. Clinical scenarios demonstrated awareness of specific DDI risks, with cautious selection of ARTAs and co-medications in line with current recommendations.

**Conclusion:**

Although limited by potential selection bias and restricted generalizability, these findings suggest that strengthening multidisciplinary collaboration could enhance patient safety and optimize therapy outcomes.

Implications for practice This international survey provides real-world insights into how clinicians from different countries manage drug–drug interactions (DDIs) in patients with prostate cancer, highlighting variability in awareness, assessment, and decision-making. By identifying gaps in risk stratification, use of interaction-checking tools, and multidisciplinary collaboration, the findings underscore the need for more standardized approaches to DDI management. These results may support the development of practical guidance and educational strategies to improve patient safety, optimize treatment selection, and enhance the clinical management of patients receiving androgen receptor-targeted agents in the context of polypharmacy.

## Introduction

Prostate cancer (PC) is predominantly a disease of older men, many of whom take multiple medications for age-related comorbidities.^1^ In this context, polypharmacy can raise the risk of drug–drug interactions (DDIs) with androgen receptor-targeted agents (ARTAs), as the Italian AIOM (Italian Association of Medical Oncology)-SIF (Italian Society of Pharmacology) position statement noted.^2^ Several studies documented a high prevalence of potential DDIs in patients with PC.^3^ In one retrospective French series of 95 men with metastatic castration-resistant PC (mCRPC) receving abiraterone acetate, 66 potential DDIs were identified in 49 patients (52%).^4^ Notably, factors like pain and poor performance status (PS) were associated with higher DDI risk, underscoring the clinical complexity of the treatment in this population.

ARTAs such as abiraterone, enzalutamide, apalutamide, or darolutamide have become central in PC treatment, and their use in polymedicated patients heightens DDI concerns.^5^^,^^6^ Abiraterone and enzalutamide can dramatically alter the metabolism of many co‑administered drugs because the first one inhibits CYP2C8 and CYP2D6, while the second one strongly induces CYP3A4 (as well as CYP2C9 and CYP2C19).^7^^,^^8^ In practical terms, enzalutamide has been shown to lower plasma levels of CYP3A4/2C9/2C19 substrates (like warfarin) while abiraterone (plus prednisone) can raise levels of CYP2D6/CYP2C8 substrates. Moreover, the literature suggests abiraterone plus prednisone may cause fewer DDIs than enzalutamide, which induces drug clearance via CYP3A4.^7^^,^^8^ Del Re et al.^7^ noted that abiraterone/prednisone and enzalutamide differ in their DDI profiles, and they stressed the need for vigilant monitoring of ARTA interactions. Even real‐world data confirm this pattern: a study of 1515 patients with no mCRPC found that 58% of apalutamide and 54% of enzalutamide patients had at least one potential DDI, compared to 5% of darolutamide.^9^ In other words, darolutamide’s more favorable pharmacokinetics translated into far fewer co-medication alerts. Also in pre‑specified and post‑hoc analyses of ARAMIS trial, researchers investigated whether concomitant medications affected pharmacokinetics of darolutamide and whether darolutamide influenced the safety of co‑administered drugs. Pharmacokinetic analysis found no significant impact of commonly used drugs on the pharmacokinetics of darolutamide, despite theoretical interaction. Interestingly, adverse event rates were similar among statin users vs non‑users, suggesting that darolutamide’s interaction with rosuvastatin did not translate into increased clinical toxicity. Overall, these results indicate that darolutamide has a low potential for clinically relevant DDIs with commonplace comedications in nmCRPC patients, supporting its favorable interaction profile in the context of polypharmacy.^10^

Key clinical drug classes, like cardiovascular medications agents, further complicate the picture. For instance, recent analyses predict numerous pharmacokinetic interactions between ARTAs and antithrombotic drugs.^11^ Abiraterone and darolutamide show little interaction with anticoagulants, but apalutamide and enzalutamide–both strong inducers of CYP2C9/2C19/3A4—may significantly alter levels of warfarin, direct‐acting oral anticoagulants (DOACs) and antiplatelets, with risks of bleeding or thrombosis. A Delphi panel of experts reached consensus that certain ARTA–anticoagulant pairs (especially involving apixaban or rivaroxaban) should be avoided or dose‐adjusted, whereas others (dabigatran, edoxaban, or warfarin) are preferred due to lower DDI risk. These guidelines highlight how clinicians are grappling with which ARTA/antithrombotic combinations are safest.^5^^,^^12^

Beyond their pharmacokinetic characterization, DDIs have a tangible clinical impact, potentially leading to reduced treatment efficacy, increased toxicity, or serious adverse events such as bleeding, cardiovascular complications, or loss of disease control. Importantly, DDIs are commonly classified according to their clinical relevance (eg, minor, moderate, or major/contraindicated), reflecting the magnitude of their impact and the need for clinical intervention (monitoring, dose adjustment, or treatment modification). Therefore, assessing not only the presence but also the severity of DDIs is crucial to properly estimate the actual risk for patients receiving ARTAs in real-world clinical practice.^2^

In this landscape, an important issue is the reliability of DDI databases and checkers. Oncologists often rely on electronic interaction‐checking tools, but studies show poor concordance between them. For common co-medications like proton pump inhibitors (PPIs) or DOACs, different DDI-checking software can give divergent alerts. In a study, investigators found significant disagreement among 5 major DDI checkers (and between European vs FDA drug labels) for PPIs, underscoring the need to standardize DDI information. Similarly, in a comparative analysis on DOACs, the level of agreement among 5 checkers was extremely low. Such discrepancies reinforce expert advice to consult multiple sources and involve clinical pharmacology specialists when evaluating potential interactions.^13^^,^^14^

National and international bodies have begun to emphasize these concerns. The Italian AIOM–SIF position statement on cancer DDIs recommends periodic medication review and multidisciplinary management in order to combine clinical and pharmacological expertise.^2^ Likewise, a recent Spanish review of mHSPC therapy underscored that “before patients are treated with an ARTA, careful assessment of potential DDI is a fundamental component in clinical decision-making.”^1^

Nevertheless, despite these recommendations and accumulating evidence, systematic data on real‐world DDI management in PC are scarce in literature. To our knowledge, no large survey has captured how oncologists in Italy and in the world actually monitor and handle DDI in patients with PC. This gap in knowledge motivates the present survey, which aims to elucidate current practices, barriers and needs in DDI management among clinicians treating PC.

## Materials and methods

The survey consisted of a self-administered, multiple-choice online questionnaire. It was developed by 5 authors (A.A.V.; S.K.; F.S.; M.C.C.; M.M.), and then reviewed and discussed by another 3 coauthors (M.D.M.; D.S.; E.Z.). Thereafter, an invitation to complete the survey was sent by e-mail to 2861 AIOM and 481 Meet-URO members from 1 December 2025 to 16 January 2026. The survey was also sent via ONCOassist mobile-app, a CE-approved oncology clinical decision support tool. Since it was originally developed in University College Cork through the Masters in E-business program in 2012, ONCOassist has received wide-scale acceptance amongst oncology clinicians globally. It was promoted by the ESMO in 2015 and is now used in more than 180 countries worldwide. The survey was sent to 19,139 registered healthcare professionals (HCPs), including physicians and nurses across various continents, and was conducted from 19 December 2025 to 31 January 2026. The questionnaire was sent in English via a push notification and pop up. Not all users received the survey due to-opt-outs, filtering criteria for HCPs, or recent contact from ONCOassist. Participants only received the survey once.

The survey sent to AIOM and Meet-URO members, in Italian language, consisted of 18 multiple choice questions. The first 5 questions were focused on the characteristics of the respondents (Part 1, Table S1 in Supplementary); other 8 questions dealt with oncological management of DDI in patients with PC (Part 2, Table S1); the last 5 questions were clinical cases, and in this section the answers were optional (Part 3, Table S1). These clinical scenarios were chosen to represent the situations clinicians most frequently face in everyday practice. In the English version submitted through the ONCOassist app, only 6 questions from Part 2 were sent (Table S2) to make the completion faster and more encouraging. Demographic and professional information were collected via the ONCOassist user’s profile, where these details were self-reported during the registration process.

Descriptive statistics were used to summarize respondents’ characteristics. Statistical analysis is described in Supplementary. For questions permitting multiple selections, percentages were calculated using the number of respondents as the denominator. Because respondents could select more than one response, percentages may exceed 100%.

## Results

Unless otherwise specified, percentages are reported for each question. For items allowing multiple responses, percentages may exceed 100% because respondents could select more than one response.

Out of 3342 invited Italian oncologists, 69 (2.1%) of them completed the survey. Considering professional society membership, most participants (43; 62.3%) belonged to both AIOM and Meet-URO, 16 (23.2%) were members of AIOM only, and 10 (14.5%) were members of Meet-URO only.

**Figure 1.**
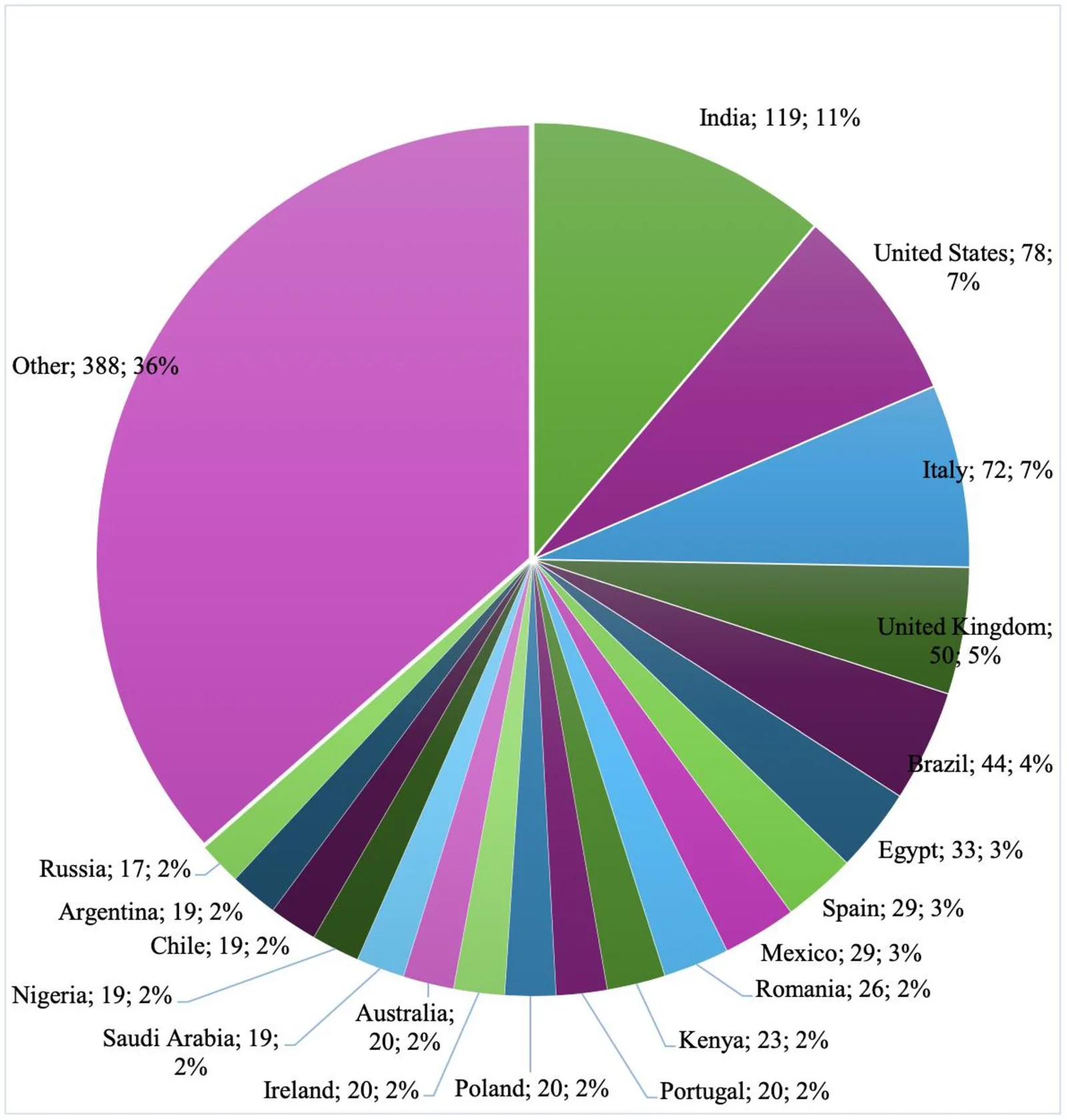
Distribution of respondents to the survey via the ONCOassist app by country.

On ONCOassist app, a total of 1064 (8.4%) recipients by different countries (Figure 1) responded to the survey, of which 791 HCPs completed all questions with a response rate stands at 6%.

Regarding professional role, most respondents were consultants/attendings (436, 41.0%) or trainee oncologists (345, 32.4%), while physician assistants, pharmacists, and oncology nurses accounted for smaller proportions of the sample (9.2%, 9.2%, and 8.0%, respectively). Two respondents reporting other professional roles were excluded from role-specific analysis, Table 1. Responses to each question of the international survey according to professional role and continent can be found in Tables S3–S13.

**Table 1.** Characteristics of respondents by continent and professional role.

Continent	*n*	**%**	Professional role	*n*	**%**
**Europe**	343	32.2	Consultant/Attending	436	41.0
**Asia**	307	28.9	Trainee oncologists	345	32.4
**North America**	142	13.3	Physician assistant	98	9.2
**Africa**	130	12.2	Pharmacist	98	9.2
**South America**	114	10.7	Oncology nurse	85	8.0
**Oceania**	28	2.6			

### Part 1 of questionnaire: respondents’ characteristics

This section has been completed only by Italian oncologists.

Regarding gender, 40 participants (58.0%) were male, while 29 (42.0%) were female. The age distribution showed that just over half of the participants (35; 50.7%) were younger than 40 years, 18 (26.1%) were between 40 and 50 years, 10 (14.5%) were between 50 and 60 years, and 6 (8.7%) were 60 years or older. In terms of geographic distribution, 24 participants (34.8%) were from the Northwestern region, 22 (31.9%) from the Central region, 13 (18.8%) from the Northeastern region, 7 (10.1%) from the Southern region, and 3 (4.3%) from the Islands. Regarding healthcare setting, the majority of participants worked in general hospitals (30; 43.5%), followed by university hospitals (23; 33.3%) and Scientific Institute for Research, Hospitalization and Healthcare (IRCCS) (14; 20.3%). Only a small number practiced in private facilities (1; 1.4%) or other settings (1; 1.4%).

### Part 2 of questionnaire: management of drug–drug interactions in PC

When asked about the relevance of DDIs in patients with PC (*Question 2.1*), 2 respondents (2.9%) considered them not relevant, 4 respondents (5.8%) considered them slightly relevant, 38 respondents (55.1%) considered them quite relevant, and 25 respondents (36.2%) considered them very relevant. Among respondents worldwide on the ONCOassist app, the relevance of DDIs in PC care was rated on a 5-point scale, ranging from 1 (not relevant) to 5 (very relevant) and reported as follows (*Question 4.1*): score 1 by 3.8% (*n* = 40); score 2 by 4.3% (*n* = 46); score 3 by 15.1% (*n* = 161); score 4 by 20.9% (*n* = 222); score 5 by 56.2% (*n* = 598). Overall, 77.1% (*n* = 820) rated DDIs as highly relevant (scores 4–5), with 56.2% assigning the highest rating (score 5).

With respect to the timing of DDI assessment (*Question 2.2*), 54 respondents (78.3%) reported evaluating potential DDI at the time of prescribing oncologic treatment for all patients, 14 respondents (20.3%) assessed interactions only in patients receiving multiple concomitant medications, and 1 respondent (1.4%) assessed interactions only after adverse events (AEs) occurred during treatment.

Respondents were then asked which resources they regularly use to assess potential DDIs in patients with PC (*Question 2.3*). Multiple responses were allowed. The most frequently used resource was online databases, such as Drugs.com and UpToDate, which were reported by 60 respondents (87.0%). Mobile applications, including ONCOassist, were used by 49 respondents (71.0%). The scientific literature was consulted by 45 respondents (65.2%), and hospital pharmacologists were regularly involved by 28 respondents (40.6%). Artificial intelligence (AI)–based tools were used less frequently, with 13 respondents (18.8%) reporting their use. Notably, 2 respondents (2.9%) indicated that they did not use any resource for DDI assessment. Among respondents worldwide on the ONCOassist app, DDIs were checked using the following resources (*Question 4.2*): online databases by 59.6% (*n* = 634); smartphone apps by 44.8% (*n* = 477); literature by 28.9% (*n* = 308); AI tools by 23.3% (*n* = 248); consultation with a pharmacologist by 23.0% (*n* = 245); and none by 3.8% (*n* = 40).

Regarding the availability of a hospital pharmacologist (*Question 2.4*), 20 respondents (29.0%) indicated that a pharmacologist was available in their center, 39 respondents (56.5%) reported that no pharmacologist was available, and 10 respondents (14.5%) stated that only external consultation was possible. Among respondents worldwide on the ONCOassist app (*n* = 899), the availability of a hospital pharmacologist at their center was reported as follows (*Question 4.3*): yes by 75.9% (*n* = 682); no by 20.5% (*n* = 184); external only by 3.8% (*n* = 34).

Among Italian respondents with access to a pharmacologist, consultation patterns varied (*Question 2.5*): 26 respondents (37.7%) consulted a pharmacologist only when patients were on more than 5 concomitant medications, 23 respondents (33.3%) consulted only in cases of discrepancies between different DDI checkers, 12 respondents (17.4%) reported never consulting a pharmacologist, and 8 respondents (11.6%) consulted a pharmacologist in all cases.

When discrepancies between different DDI checkers were encountered, respondents reported different management strategies (*Question 2.6*). Specifically, 23 respondents (33.3%) requested a pharmacological opinion, 21 respondents (30.4%) consulted the scientific literature, 19 respondents (27.5%) considered the most severe interaction reported, and 6 respondents (8.7%) relied on personal clinical judgment. Among respondents worldwide on the ONCOassist app (*n* = 899), conflicting DDI results were resolved as follows (*Question 4.4*): pharmacology consult by 33.9% (*n* = 305); literature review by 31.4% (*n* = 282); clinical judgment by 26.4% (*n* = 237); and following the most severe reported interaction by 8.6% (*n* = 77).

The main obstacles to systematic evaluation of DDIs (*Question 2.7*) were identified as uncertainty regarding the reliability of sources, reported by 24 respondents (34.8%); lack of time, reported by 19 respondents (27.5%); lack of reliable tools, reported by 15 respondents (21.7%); and lack of specific training, reported by 11 respondents (15.9%). Among respondents worldwide on the ONCOassist app, the main barriers to systematic DDI evaluation were reported as follows (*Question 4.5*): lack of time by 39.7% (*n* = 422); uncertain reliability of sources by 25.1% (*n* = 267); lack of reliable tools by 21.4% (*n* = 228) and lack of oncologist training by 20.6% (*n* = 219).

Regarding potential solutions to improve DDI management (*Question 2.8*), 40 respondents (58.0%) considered access to an on-demand pharmacologist as the most effective solution, 12 respondents (17.4%) suggested training campaigns, 11 respondents (15.9%) recommended dedicated sections on institutional or company websites, and 6 respondents (8.7%) proposed in-house or institutional courses. Among respondents worldwide on the ONCOassist app, the most effective ways to increase DDI awareness were reported as follows (*Question 4.6*): institutional training by 38.3% (*n* = 408); pharmacologist access by 33.2% (*n* = 353); educational events by 30.5% (*n* = 324); and institutional web resources by 26.0% (*n* = 277).

Significant geographic variations were observed in the perceived relevance of DDIs in PC care (*P *< .001) (Table S4). The highest proportion of respondents reporting “score 5” was observed in South America (75.4%); pairwise comparisons confirmed significantly higher proportions of maximal relevance scores in South America (Table 2). Accordingly, in ordinal logistic regression analysis, respondents from South America showed significantly higher odds of reporting higher relevance scores (OR 2.27, 95% CI 1.44-3.68; *P *< .001), while those from Asia reported lower perceived relevance (OR 0.72, 95% CI 0.54-0.97; *P *= .028), compared with Europe respondents (reference). The availability of a hospital pharmacologist varied significantly across continents (*P *= .017) (Table S7), with differences observed particularly between Asia and Oceania (Table 3). Differences across continents were also observed in the management of conflicting DDI results (*P *< .001), Table S9: respondents from Asia more frequently reported consulting the literature compared with those from Europe (38.5% vs 25.3%), whereas clinicians from Africa more frequently reported pharmacology consultation compared with those from Asia (45.6% vs 25.4%, *P *= .002) (Table 2). Geographical variations were also observed in perceived barriers to DDI evaluation (*P *= .034): lack of time was reported more frequently by European respondents compared with African respondents (43.3% vs 26.5%, *P *= .004; Table 2).

**Table 2.** Significant differences in specific response options.

Question	Option	Group comparison	**%** Group 1	**%** Group 2	Adjusted *P*
**DDI relevance by continent**	Score 5	Asia vs South America	50.5	75.4	<.001
	Score 5	Europe vs South America	56.3	75.4	.003
**Check DDIs by role**	Pharmacologist	Oncology nurse vs Trainee oncologists	20.8	10.6	.004
	Pharmacologist	Consultant vs Oncology nurse	12.0	28.0	.019
**Conflicting DDI results by continent**	Literature review	Asia vs Europe	38.5	25.3	.014
	Literature review	Europe vs South America	25.3	42.3	.021
	Pharmacology consult	Africa vs Asia	45.6	25.4	.002
**Conflicting DDI results by role**	Literature review	Oncology nurse vs Trainee oncologists	8.7	36.1	<.001
	Pharmacology consult	Consultant vs Oncology nurse	29.6	66.7	<.001
**Barrier by continent**	Lack of time	Africa vs Europe	26.5	43.3	.004

**Table 3.** Significant pairwise comparisons between groups.

Question	Comparison	Adjusted *P* value
**DDI relevance by continent**	Asia vs South America	.001
	Oceania vs South America	.007
	Africa vs South America	.012
	Europe vs South America	.035
**Hospital pharmacologist availability**	Asia vs Oceania	.009
**Conflicting DDI results by continent**	Africa vs Asia	<.001
**Conflicting DDI results by role**	Consultant vs Oncology nurse	<.001
	Oncology nurse vs Trainee oncologist	<.001
	Oncology nurse vs Pharmacist	<.001
	Oncology nurse vs Physician assistant	.003
**Barrier by continent**	Africa vs Europe	.032

Overall, the perceived relevance of DDIs did not significantly differ across professional roles (Table S3). However, when evaluating high DDI awareness using logistic regression, pharmacists showed significantly higher odds compared with consultants/attendings (reference) (OR 1.86, 95% CI 1.06-3.47; *P *= .040). Differences across professional roles were also observed in the strategies used to check DDIs (*P *= .045) (Table S5). In particular, oncology nurses reported more frequent consultation with pharmacologists compared with trainee oncologists and consultant (20.8% vs 10.6% vs 12.0%, *P *= .004 and .019, respectively; Table 2). Similarly, differences emerged in how clinicians managed conflicting DDI results (*P *< .001; Table S8). Oncology nurses showed significantly different decision patterns compared with other professional groups, Tabel 3: they reported substantially higher reliance on pharmacology consultation (66.7%) compared with consultants (29.6%) and trainees (32.3%), *P *< .001. Conversely, literature review was more frequently reported by trainee oncologists compared with oncology nurses (36.1% vs 8.7%, *P *< .001; Table 2).

### Part 3 of questionnaire: exploratory clinical scenarios among Italian respondents only

Part 3 of the survey was completed on a voluntary basis exclusively by Italian oncologists. A summary of the responses is presented in Table 4.

**Table 4.** Summary of responses by Italian oncologists to clinical scenarios (Part 3).

Clinical scenario	Options	*N*. responses/*N*. respondents (**%**)
**3.1 A patient with prostate cancer receiving a DOACs (e.g., apixaban, rivaroxaban) needs to start an ARTA. How do you proceed?**		
	Prefer abiraterone over enzalutamide due to lower interaction risk	15/66 (22.7)
	Prefer enzalutamide over abiraterone due to lower interaction risk	5/66 (7.6)
	Choose ARTA regardless of the anticoagulant, given the low interaction risk between the 2 drug classes	10/66 (15.1)
	Request hematology (or vascular medicine) consultation to adjust/monitor dose or change anticoagulant before starting any ARTA	31/66 (47)
	Request pharmacological consultation	5/66 (7.6)
**3.2 A patient receiving abiraterone acetate + prednisone requires a PPI. How do you proceed?**		
	Start or continue PPI therapy without concern, as there is no strong evidence of high-risk interaction with abiraterone	28/65 (43)
	Prefer certain PPIs over others due to different levels of interaction with abiraterone + prednisone	20/65 (30.8)
	Advise the patient to avoid any PPI while on abiraterone due to interaction risk	12/65 (18.5)
	Request pharmacological consultation	5/65 (7.7)
**3.3 A patient receiving abiraterone, enzalutamide, darolutamide, or apalutamide requires dual antiplatelet therapy with acetylsalicylic acid (ASA) + ticagrelor. How do you proceed?**		
	Consider the most severe interaction and prefer abiraterone or darolutamide over enzalutamide or apalutamide due to lower interaction risk	20/65 (30.8)
	Withhold ARTA therapy	1/65 (1.5)
	Choose ARTA regardless of antiplatelet therapy	5/65 (7.7)
	Request cardiology consultation to modify antiplatelet therapy (eg, ASA alone or ASA + clopidogrel) before starting any ARTA	32/65 (49.2)
	Request pharmacological consultation	7/65 (10.8)
**3.4 A patient receiving abiraterone, enzalutamide, darolutamide, or apalutamide requires oral antibiotic therapy. How do you proceed?**		
	If multiple antibiotics are options, choose the one with the lowest or no interaction with ARTA therapy	38/66 (57.6)
	Discontinue ARTA only if the prescribed antibiotic has major interactions	13/66 (19.7)
	Temporarily discontinue ARTA regardless of interaction risk	7/66 (10.6)
	Choose the antibiotic regardless of potential interactions and continue ARTA therapy	3/66 (4.5)
	Request pharmacological consultation	5/66 (7.6)
**3.5 A patient receiving fentanyl needs to start enzalutamide. How do you proceed?**		
	Request modification of pain management therapy before starting enzalutamide	29/65 (44.6)
	Start enzalutamide given the absence/minimal interaction risk	36/65 (55.4)

## Discussion

Our international survey confirms that DDIs in PC care are widely recognized as clinically important, confirming that awareness of DDI risk is high across settings.

Despite both groups agreed that DDIs are a relevant concern, the collected data highlight some interesting differences between practice patterns of Italian oncologists and the international cohort. These observations should be interpreted cautiously, as the two cohorts were not designed to be directly comparable. Differences in sampling framework, professional composition, survey language, and questionnaire structure may have influenced respondents’ answers. Therefore, comparisons between the Italian and ONCOassist cohorts should be regarded as exploratory and hypothesis-generating.

First of all, our results show geographic and professional variations in the perception and management of DDIs in PC care. DDIs were consistently rated as highly relevant worldwide, with particularly high scores reported in South America, although this finding may be influenced by sampling and survey bias. Observed regional differences likely reflect disparities in healthcare system structure, drug availability, and access to multidisciplinary pharmacological support. Professional roles also influenced DDI management strategies, with variations in reliance on pharmacology consultation versus literature-based decision-making. Overall, heterogeneous approaches to DDIs persist and may have important implications for patient safety in complex oncological treatment settings.

In both cohorts of respondents, digital resources represented the most commonly used tools for DDIs: online databases and smartphone apps were the most frequently consulted resources, confirming the central role of easily accessible electronic references in routine clinical decision-making. Italian respondents reported heavier use of traditional literature searches. Interestingly, the use of AI–based tools remained relatively limited in both cohorts, suggesting that AI-assisted DDI assessment is still in an early phase of clinical implementation. This may reflect limited familiarity with such tools and uncertainty regarding their reliability.

A notable difference emerged in the availability of hospital pharmacologists. In Italian cohort, more than half of respondents indicated that no pharmacologist was available at their center, while the majority of ONCOassist respondents (75.9%) reported the availability of a hospital pharmacologist. This discrepancy may reflect structural differences in healthcare organization and multidisciplinary support across healthcare systems. Limited access to pharmacological expertise among Italian clinicians could potentially increase the reliance on digital tools and self literature consultation, which indeed was often considered to resolve DDI discrepancies. However, despite differences to access hospital pharmacologist, in both cohorts, the most common approach to resolve DDI discrepancies was to seek pharmacologist consultation. Interestingly, reliance on personal clinical judgment was reported more frequently in the international cohort compared with the Italian respondents, whereas Italian clinicians more often reported considering the most severe interaction identified. These contrasts may reflect cultural and healthcare-system differences as well as the fact that the Italian survey was limited to AIOM/Meet-URO members while the ONCOassist sample represents a tech-selected group of app users. However, our survey underscores a known problem: different checkers may not agree. This lack of standardization is precisely why our respondents often turned to pharmacologist or literature when app alerts conflicted.

The analysis of perceived barriers further highlights important challenges in the systematic assessment of DDIs. Among Italian respondents, the most frequently reported barrier was uncertainty regarding the reliability of available sources followed by lack of time, whereas in the global cohort lack of time emerged as the primary limitation. Notably, in both populations, at least 20% of respondents reported the lack of reliable tools as a relevant barrier, underscoring the perceived limitations of currently available DDI checkers. This finding reinforces the need for more standardized and validated systems for DDI assessment. Reassuringly, the proportion of respondents reporting insufficient training in DDI management was relatively low in both cohorts. This observation is consistent with the high level of awareness of the relevance of DDIs in PC care reported in the survey. Taken together, these findings suggest that the main challenges in DDI management may not primarily be limited clinician knowledge, but rather from practical constraints such as the lack of reliable tools and time. Therefore, both structural and informational barriers contribute to suboptimal DDI management.

Eventually, the perceived solutions to improve DDI management also differed between the two cohorts. Italian respondents most frequently identified access to a pharmacologist as the most effective strategy, likely reflecting the limited availability of pharmacological support previously reported within this group. In contrast, the global cohort more commonly emphasized the role of institutional training and education initiatives. The observed differences in proposed strategies to overcome barriers may indicate that Italian clinicians give particular value to institutional support mechanisms, such as dedicated pharmacy services, whereas their global peers appear to place greater importance on strategies to increase individual competence in DDI management.

Comparing our survey clinical vignettes—where the responses reflected an appropriately cautious approach—illustrates some points.^2^ The findings from the clinical vignettes should be interpreted separately from the international survey results. These scenarios were optional and completed exclusively by a subset of Italian respondents; therefore, they cannot be generalized to the global ONCOassist cohort. Respondents showed knowledge of known interaction patterns among ARTAs and co-medications. For example, in the vignette of a patient on a DOAC, 22% favored abiraterone over enzalutamide and none enzalutamide, reflecting the fact that enzalutamide strongly induces CYP3A4/2C9/2C19 (lowering warfarin levels) while abiraterone mainly affects CYP2D6/CYP2C8. A recent consensus urges avoidance of apixaban and rivaroxaban in ARTA-treated patients and favors dabigatran, edoxaban or warfarin (which can be monitored) because they have lower interaction risk.^12^ Our respondents’ cautious approach—45% did not choose an ARTA at all but instead sought specialist input—is in line with these guidelines. Similarly, when an ARTA patient required antiplatelet therapy, almost half of oncologists deferred to cardiology rather than starting ARTA without adjustment. This again is concordant with expert recommendations: apalutamide and abiraterone can increase active metabolites of ticagrelor and clopidogrel, and cardiology consultation or choosing antiplatelets with fewer interactions is reccomended.^14^ For antibiotic interactions, most respondents (55%) would pick an agent with the least ARTA interaction and continue therapy, emphasizing the need to choose antibiotics that avoid CYP3A4 or QTc interactions.^11^ Even in the PPI scenario, respondents were divided: 41% said PPIs were safe with abiraterone, while 17% would avoid them. A recent pooled analysis found that concomitant PPI use was associated with significantly worse survival on abiraterone, suggesting some justification for caution (and supporting the 17% who avoid PPIs).^13^ Overall, these responses demonstrate that many oncologists are aware of specific DDI risks (favoring low-interaction ARTAs or consulting specialists) and that their concerns are broadly aligned with current expert advice.

This survey have both strengths and limitations. The strengths: (1) recent data and (2) sample of the respondents (first survey to capture both an Italian and a global sample, strengthening the generalizability of the results).

The limitations: (1) relatively low response rates, particularly in the Italian cohort, may have introduced substantial selection bias. Participants may have been more interested in DDI management than non-responders, potentially leading to an overestimation of awareness and engagement with this issue; (2) Italian sample skewed younger (51% under 40) and hospital-based, while ONCOassist respondents are users of smartphone technology and may not mirror all global practitioners; (3) findings based on oncologists’ self-reports (to obtain more definitive insights, a prospective or retrospective observational study, for example, the ongoing MITO34 trial,^15^ using patient-level data would be necessary); (4) Part 3 was optional and completed only by few Italian respondents limiting comparability across international sample; (5) the term “pharmacologist” may introduce ambiguity and potential bias because this role is defined differently across countries (eg, often corresponding to pharmacists in the United Kingdom), potentially affecting the interpretation of questions among an international respondent pool; (6) the lack of granularity in clinicians’ responses regarding the type and classification of interactions should be acknowledged, along with the heterogeneity between the two cohorts (Italian oncology specialists vs ONCOassist mixed group of HCPs); (7) the cross-sectional design of this survey provides a snapshot of clinicians’ perceptions at a single point in time, not fully capturing how awareness and management strategies evolve; and (8) the Italian and ONCOassist surveys were not fully equivalent in design. The two cohorts differed with respect to respondent characteristics, language of administration, survey content, and availability of clinical vignettes. Consequently, any comparison between cohorts should be interpreted as exploratory rather than confirmatory.

## Conclusion

Despite the limited generalizability due to low response rates and potential selection bias, this survey highlights reveal persistent gaps (heterogeneity in access to pharmacologist support, uncertainty regarding reliable information sources, inconsistent strategies for DDI mitigation) emphasizing the urgent need for robust system-level solutions (clear demand for pharmacologist support, routine medication reconciliation). For centers lacking on-site pharmacologist, telepharmacy consults or hotlines could help meet the expressed need for “on-demand” expertise. Professional societies and institutions should reinforce DDI modules in continuing education. Validated tools/DDI databases (powered by AI or pharmacogenomic data) should be created. Finally, future research should measure the real-world clinical impact of DDIs on patient outcomes and toxicities.^15^

## Supplementary Material

oyag360_Supplementary_Data

## Data Availability

The data underlying this article are available in the article and in its online supplementary material.
