# Supplementary material for: Global management of drug–drug interaction in patients with prostate cancer: international survey by Meet-URO group and ONCOassist
Source: Oncologist. 2026 Sep 16;31(10):oyag360. doi: 10.1093/oncolo/oyag360 (PMC13596776; doi:10.1093/oncolo/oyag360)
Supplement: Supplementary file 1 [file oyag360_supplementary_data.docx]

**Supplementary materials**

*“Global management of drug-drug interaction in patients with prostate cancer: international survey by Mee-URO group and ONCOassist”*

| **Statistical analysis** | **p.2** |
| --- | --- |
| **Table S1.** Questions of survey translated in English language sent to AIOM and Meet-URO members | **p.3-5** |
| **Table S2.** Questions in English language of survey sent via ONCOassist app. | **p.6** |
| **Table S3**. How relevant are DDIs in prostate cancer care? Responses stratified by professional role | **p.7** |
| **Table S4**. How relevant are DDIs in prostate cancer care? Responses stratified by continent | **p.8** |
| **Table S5**. How do you check DDIs? Responses stratified by professional role | **p.9** |
| **Table S6**. How do you check DDIs? Responses stratified by continent | **p.10** |
| **Table S7**. Is a hospital pharmacologist available at your center? Responses stratified by continent | **p.11** |
| **Table S8.** How do you resolve conflicting DDI results? Responses stratified by professional role | **p.12** |
| **Table S9**. How do you resolve conflicting DDI results? Responses stratified by continent | **p.13** |
| **Table S10**. Main barrier to systematic DDI evaluation; responses stratified by professional role | **p.14** |
| **Table S11.** Main barrier to systematic DDI evaluation; responses stratified by continent | **p.15** |
| **Table S12.** Most effective way to increase DDI awareness; responses stratified by professional role | **p.16** |
| **Table S13**. Most effective way to increase DDI awareness; responses stratified by continent | **p.17** |

**Statistical analysis**

Differences in response distributions across continents and professional roles were evaluated using Pearson’s chi-squared test and Kruskal–Wallis test for ordinal variable. Pairwise comparisons between groups and answers were also performed. To account for multiple testing, *p* values were adjusted using the Holm method, and only adjusted *p* values were reported for these comparisons. Ordinal logistic regression models were generated and separate models were constructed to evaluate the association between DDI relevance score and professional role or continent. Odds ratios (ORs) with 95% confidence intervals (CIs) were obtained by exponentiating the model coefficients. Statistical significance was set at *p* < 0.05. All analyses were conducted using R software (version 4.4.2).

**Table S1.** Questions of survey translated in English language sent to AIOM and Meet-URO members

**Part 1 – Respondent Characteristics**

**1.1 Gender**
☐ Male
☐ Female

**1.2 Age**
☐ Under 40 years
☐ Between 40 and 50 years
☐ Between 50 and 60 years
☐ Over 60 years

**1.3 Geographic Area**
☐ Northwestern Italy
☐ Northeastern Italy
☐ Central Italy
☐ Southern Italy
☐ Islands (Sicily and Sardinia)

**1.4 Healthcare Facility Where You Work**
☐ General hospital
☐ University hospital
☐ IRCCS (Scientific Institute for Research, Hospitalization and Healthcare)
☐ Private hospital
☐ Other

**1.5 Professional Role**
☐ Hospital-based oncologist
☐ University oncologist
☐ Oncology resident
☐ Private practitioner

**Part 2 – Management of Drug–Drug Interactions in Prostate Cancer**

**2.1 How relevant do you consider the issue of drug–drug interactions (DDIs) in patients with prostate cancer?**
☐ Not relevant
☐ Slightly relevant
☐ Neutral
☐ Fairly relevant
☐ Very relevant

**2.2 At which stage do you assess potential drug interactions?**
☐ At the time of prescribing oncologic treatment, in all patients
☐ At the time of prescribing oncologic treatment, only in patients taking multiple concomitant medications (e.g., more than 5)
☐ Only in case of adverse events during treatment

**2.3 Which resources do you regularly use to assess drug interactions? (Select all that apply)**
☐ Online databases (e.g., Drugs.com, UpToDate, etc.)
☐ Scientific literature
☐ Mobile apps (e.g., INTERCheck, OncoAssist, etc.)
☐ Artificial intelligence–based tools
☐ Hospital pharmacologist
☐ None

**2.4 Is a hospital pharmacologist available in your center?**
☐ Yes
☐ No
☐ Not internally, but external consultation is available

**2.5 If a pharmacologist is available, when do you consult (or would you consult) them?**
☐ Never
☐ Always
☐ Only in cases of polypharmacy (e.g., more than 5 concomitant drugs)
☐ Only in case of discrepancies between different drug-interaction checkers

**2.6 In case of discrepancies between different drug-interaction checkers, how do you manage them?**
☐ Rely on my own clinical judgment and experience
☐ Consult the scientific literature
☐ Request pharmacological consultation
☐ Consider the most severe interaction reported among the different tools

**2.7 In your opinion, what is the most relevant barrier to a systematic evaluation of DDIs? (Select the main one)**
☐ Lack of time
☐ Uncertainty regarding reliability of sources and data
☐ Lack of specific training in oncology
☐ Lack of reliable resources/tools

**2.8 In your opinion, what would be the most effective solution to improve awareness of drug interactions and support clinicians in daily practice? (Select the main one)**
☐ In-house training courses
☐ Educational events/campaigns
☐ On-demand pharmacologist or pharmacology consultation
☐ Dedicated sections available on institutional/company websites

**Part 3 – Clinical Scenarios**

*From this point onward, responses are optional: the following section includes 5 common clinical scenarios encountered in daily practice (estimated completion time: 5 minutes). If you prefer not to complete them, you may proceed directly to the end of the questionnaire.*

**3.1 A patient with prostate cancer receiving a direct oral anticoagulant (e.g., apixaban, rivaroxaban) needs to start an ARPI. How do you proceed?**
☐ Prefer abiraterone over enzalutamide due to lower interaction risk
☐ Prefer enzalutamide over abiraterone due to lower interaction risk
☐ Choose ARPI regardless of the anticoagulant, given the low interaction risk between the two drug classes
☐ Request hematology (or vascular medicine) consultation to adjust/monitor dose or change anticoagulant before starting any ARPI
☐ Request pharmacological consultation

**3.2 A patient receiving abiraterone acetate + prednisone requires a proton pump inhibitor (PPI). How do you proceed?**
☐ Start or continue PPI therapy without concern, as there is no strong evidence of high-risk interaction with abiraterone
☐ Prefer certain PPIs over others due to different levels of interaction with abiraterone + prednisone
☐ Advise the patient to avoid any PPI while on abiraterone due to interaction risk
☐ Request pharmacological consultation

**3.3 A patient receiving abiraterone, enzalutamide, darolutamide, or apalutamide requires dual antiplatelet therapy with acetylsalicylic acid (ASA) + ticagrelor. How do you proceed?**
☐ Consider the most severe interaction and prefer abiraterone or darolutamide over enzalutamide or apalutamide due to lower interaction risk
☐ Withhold ARPI therapy
☐ Choose ARPI regardless of antiplatelet therapy
☐ Request cardiology consultation to modify antiplatelet therapy (e.g., ASA alone or ASA + clopidogrel) before starting any ARPI
☐ Request pharmacological consultation

**3.4 A patient receiving abiraterone, enzalutamide, darolutamide, or apalutamide requires oral antibiotic therapy. How do you proceed?**
☐ If multiple antibiotics are options, choose the one with the lowest or no interaction with ARPI therapy
☐ Discontinue ARPI only if the prescribed antibiotic has major interactions
☐ Temporarily discontinue ARPI regardless of interaction risk
☐ Choose the antibiotic regardless of potential interactions and continue ARPI therapy
☐ Request pharmacological consultation

**3.5 A patient receiving fentanyl needs to start enzalutamide. How do you proceed?**
☐ Request modification of pain management therapy before starting enzalutamide
☐ Start enzalutamide given the absence/minimal interaction risk

**Table S2.** Questions in English language of survey sent via ONCOassist app.

**4.1 How relevant are drug-drug interactions (DDIs) in prostate cancer care?**
☐ Score 1 - Not relevant
☐ Score 2 - Slightly relevant
☐ Score 3 - Neutral
☐ Score 4 - Fairly relevant
☐ Score 5 - Very relevant

**4.2 How do you check DDIs? (Select all that apply)**
☐ Online databases (e.g., Drugs.com, UpToDate, etc.)
☐ Literature
☐ Smartphone apps
☐ AI tools
☐ Pharmacologist
☐ None

**4.3 Is a hospital pharmacologist available at your center?**
☐ Yes
☐ No
☐ External only

**4.4 How do you resolve conflicting DDI results?**
☐ Clinical Judgement
☐ Follow the most severe interaction reported

☐ Literature Review
☐ Pharmacology Consult

**4.5 Main barrier to systematic DDI evaluation (Select all that apply)**
☐ Lack of time
☐ Uncertain reliability of sources
☐ Lack of oncologist training
☐ Lack of reliable tools

**4.6 Most effective way to increase DDI awareness (Select all that apply)**
☐ Institutional training
☐ Educational events
☐ Pharmacologist access
☐ Institutional web resources

**Table S3**. How relevant are DDIs in prostate cancer care? Responses stratified by professional role

| Relevance | Consultant/Attending n (%) | Trainee oncologists n (%) | Physician assistant n (%) | Pharmacist n (%) | Oncology nurse n (%) | p value |
| --- | --- | --- | --- | --- | --- | --- |
| 1 | 19 (4.3) | 11 (3.2) | 3 (3.1) | 3 (3.1) | 4 (4.7) | 0.895 |
| 2 | 19 (4.3) | 16 (4.6) | 4 (4.1) | 3 (3.1) | 4 (4.7) |  |
| 3 | 72 (16.4) | 52 (15.0) | 16 (16.3) | 9 (9.2) | 11 (12.9) |  |
| 4 | 97 (22.1) | 72 (20.8) | 17 (17.3) | 23 (23.5) | 13 (15.3) |  |
| 5 | 231 (52.7) | 195 (56.4) | 58 (59.2) | 60 (61.2) | 53 (62.4) |  |

**Table S4**. How relevant are DDIs in prostate cancer care? Responses stratified by continent

| Relevance | Africa n (%) | Asia n (%) | Europe n (%) | North America n (%) | Oceania n (%) | South America n (%) | p value |
| --- | --- | --- | --- | --- | --- | --- | --- |
| 1 | 7 (5.3) | 14 (4.5) | 14 (4.1) | 4 (2.8) | 1 (3.6) | 0 (0.0) | **< 0.001** |
| 2 | 8 (6.1) | 18 (5.8) | 8 (2.3) | 6 (4.2) | 3 (10.7) | 3 (2.6) |  |
| 3 | 26 (19.8) | 59 (19.1) | 44 (12.8) | 18 (12.7) | 4 (14.3) | 10 (8.8) |  |
| 4 | 22 (16.8) | 62 (20.1) | 84 (24.5) | 29 (20.4) | 10 (35.7) | 15 (13.2) |  |
| 5 | 68 (51.9) | 156 (50.5) | 193 (56.3) | 85 (59.9) | 10 (35.7) | 86 (75.4) |  |

**Table S5**. How do you check DDIs? Responses stratified by professional role

| Resource | Consultant/Attending n (%) | Trainee oncologists n (%) | Physician assistant n (%) | Pharmacist n (%) | Oncology nurse n (%) | p value |
| --- | --- | --- | --- | --- | --- | --- |
| Online databases | 257 (32.4) | 205 (33.4) | 47 (28.7) | 74 (35.9) | 51 (29.5) | **0.045** |
| Literature | 128 (16.1) | 90 (14.7) | 27 (16.5) | 34 (16.5) | 28 (16.2) |  |
| Smartphone apps | 194 (24.4) | 162 (26.4) | 39 (23.8) | 47 (22.8) | 34 (19.7) |  |
| AI tools | 102 (12.8) | 83 (13.5) | 25 (15.2) | 18 (8.7) | 20 (11.6) |  |
| Pharmacologist | 95 (12.0) | 65 (10.6) | 18 (11.0) | 31 (15.0) | 36 (20.8) |  |
| None | 18 (2.3) | 8 (1.3) | 8 (4.9) | 2 (1.0) | 4 (2.3) |  |

**Table S6**. How do you check DDIs? Responses stratified by continent

| Resource | Africa n (%) | Asia n (%) | Europe n (%) | North America n (%) | Oceania n (%) | South America n (%) | p value |
| --- | --- | --- | --- | --- | --- | --- | --- |
| Online databases | 74 (28.1) | 177 (30.7) | 210 (36.3) | 85 (32.1) | 22 (44.0) | 66 (30.0) | 0.162 |
| Literature | 42 (16.0) | 95 (16.5) | 86 (14.9) | 40 (15.1) | 6 (12.0) | 39 (17.7) |  |
| Smartphone apps | 68 (25.9) | 131 (22.7) | 145 (25.1) | 64 (24.2) | 11 (22.0) | 58 (26.4) |  |
| AI tools | 31 (11.8) | 90 (15.6) | 60 (10.4) | 32 (12.1) | 3 (6.0) | 32 (14.5) |  |
| Pharmacologist | 40 (15.2) | 66 (11.5) | 67 (11.6) | 40 (15.1) | 8 (16.0) | 24 (10.9) |  |
| None | 8 (3.0) | 17 (3.0) | 10 (1.7) | 4 (1.5) | 0 (0.0) | 1 (0.5) |  |

**Table S7**. Is a hospital pharmacologist available at your center? Responses stratified by continent

| Availability | Africa n (%) | Asia n (%) | Europe n (%) | North America n (%) | Oceania n (%) | South America n (%) | p value |
| --- | --- | --- | --- | --- | --- | --- | --- |
| Yes | 88 (77.2) | 189 (73.0) | 217 (76.1) | 88 (72.7) | 18 (75.0) | 82 (84.5) | **0.017** |
| No | 20 (17.5) | 67 (25.9) | 54 (18.9) | 27 (22.3) | 3 (12.5) | 13 (13.4) |  |
| External only | 6 (5.3) | 3 (1.2) | 14 (4.9) | 6 (5.0) | 3 (12.5) | 2 (2.1) |  |

**Table S8.** How do you resolve conflicting DDI results? Responses stratified by professional role

| Strategy | Consultant/Attending n (%) | Trainee oncologists n (%) | Physician assistant n (%) | Pharmacist n (%) | Oncology nurse n (%) | p value |
| --- | --- | --- | --- | --- | --- | --- |
| Clinical judgement | 117 (31.5) | 65 (22.1) | 18 (21.7) | 23 (28.4) | 14 (20.3) | **< 0.001** |
| Follow the most severe interaction reported | 31 (8.3) | 28 (9.5) | 8 (9.6) | 7 (8.6) | 3 (4.3) |  |
| Literature review | 114 (30.6) | 106 (36.1) | 27 (32.5) | 27 (33.3) | 6 (8.7) |  |
| Pharmacology consult | 110 (29.6) | 95 (32.3) | 30 (36.1) | 24 (29.6) | 46 (66.7) |  |

**Table S9**. How do you resolve conflicting DDI results? Responses stratified by continent

| Strategy | Africa n (%) | Asia n (%) | Europe n (%) | North America n (%) | Oceania n (%) | South America n (%) | p value |
| --- | --- | --- | --- | --- | --- | --- | --- |
| Clinical judgement | 20 (17.5) | 76 (29.2) | 84 (29.5) | 29 (24.0) | 10 (41.7) | 18 (18.6) | **< 0.001** |
| Follow the most severe interaction reported | 15 (13.2) | 18 (6.9) | 27 (9.5) | 9 (7.4) | 2 (8.3) | 6 (6.2) |  |
| Literature review | 27 (23.7) | 100 (38.5) | 72 (25.3) | 38 (31.4) | 4 (16.7) | 41 (42.3) |  |
| Pharmacology consult | 52 (45.6) | 66 (25.4) | 102 (35.8) | 45 (37.2) | 8 (33.3) | 32 (33.0) |  |

**Table S10**. Main barrier to systematic DDI evaluation; responses stratified by professional role

| Barrier | Consultant/Attending n (%) | Trainee oncologists n (%) | Physician assistant n (%) | Pharmacist n (%) | Oncology nurse n (%) | p value |
| --- | --- | --- | --- | --- | --- | --- |
| Lack of time | 187 (40.0) | 144 (37.2) | 32 (36.8) | 30 (27.5) | 29 (35.4) | 0.382 |
| Uncertain reliability of sources | 106 (22.6) | 97 (25.1) | 18 (20.7) | 23 (21.1) | 22 (26.8) |  |
| Lack of oncologist training | 83 (17.7) | 71 (18.3) | 22 (25.3) | 28 (25.7) | 13 (15.9) |  |
| Lack of reliable tools | 92 (19.7) | 75 (19.4) | 15 (17.2) | 28 (25.7) | 18 (22.0) |  |

**Table S11.** Main barrier to systematic DDI evaluation; responses stratified by continent

| Barrier | Africa n (%) | Asia n (%) | Europe n (%) | North America n (%) | Oceania n (%) | South America n (%) | p value |
| --- | --- | --- | --- | --- | --- | --- | --- |
| Lack of time | 43 (26.5) | 110 (33.6) | 162 (43.3) | 53 (37.3) | 13 (48.1) | 41 (39.4) | **0.034** |
| Uncertain reliability of sources | 39 (24.1) | 85 (26.0) | 78 (20.9) | 38 (26.8) | 7 (25.9) | 20 (19.2) |  |
| Lack of oncologist training | 40 (24.7) | 57 (17.4) | 74 (19.8) | 22 (15.5) | 3 (11.1) | 23 (22.1) |  |
| Lack of reliable tools | 40 (24.7) | 75 (22.9) | 60 (16.0) | 29 (20.4) | 4 (14.8) | 20 (19.2) |  |

**Table S12.** Most effective way to increase DDI awareness; responses stratified by professional role

| Strategy | Consultant/Attending n (%) | Trainee oncologists n (%) | Physician assistant n (%) | Pharmacist n (%) | Oncology nurse n (%) | p value |
| --- | --- | --- | --- | --- | --- | --- |
| Institutional training | 162 (28.3) | 145 (31.5) | 28 (27.2) | 43 (33.6) | 29 (30.5) | 0.902 |
| Educational events | 143 (25.0) | 106 (23.0) | 25 (24.3) | 26 (20.3) | 24 (25.3) |  |
| Pharmacologist access | 142 (24.8) | 118 (25.6) | 29 (28.2) | 35 (27.3) | 28 (29.5) |  |
| Institutional web resources | 125 (21.9) | 92 (20.0) | 21 (20.4) | 24 (18.8) | 14 (14.7) |  |

**Table S13**. Most effective way to increase DDI awareness; responses stratified by continent

| Strategy | Africa n (%) | Asia n (%) | Europe n (%) | North America n (%) | Oceania n (%) | South America n (%) | p value |
| --- | --- | --- | --- | --- | --- | --- | --- |
| Institutional training | 75 (35.0) | 125 (31.6) | 115 (26.6) | 50 (29.1) | 10 (33.3) | 33 (27.7) | 0.605 |
| Educational events | 50 (23.4) | 97 (24.6) | 105 (24.3) | 36 (20.9) | 4 (13.3) | 32 (26.9) |  |
| Pharmacologist access | 45 (21.0) | 96 (24.3) | 119 (27.5) | 54 (31.4) | 8 (26.7) | 31 (26.1) |  |
| Institutional web resources | 44 (20.6) | 77 (19.5) | 93 (21.5) | 32 (18.6) | 8 (26.7) | 23 (19.3) |  |
